# Supplementary material for: The interactome of intact mitochondria by cross-linking mass spectrometry provides evidence for coexisting respiratory supercomplexes
Source: Mol Cell Proteomics. 2017 Dec 8;17(2):216–32. doi: 10.1074/mcp.RA117.000470 (PMC5795388; doi:10.1074/mcp.RA117.000470)
Supplement: Supplemental Data [file supp_RA117.000470_133922_0_supp_23978_fzffwf.zip › spectra_annotation/mito_DR_spectra_annotation/221-1-4-1-4-1.pdf]

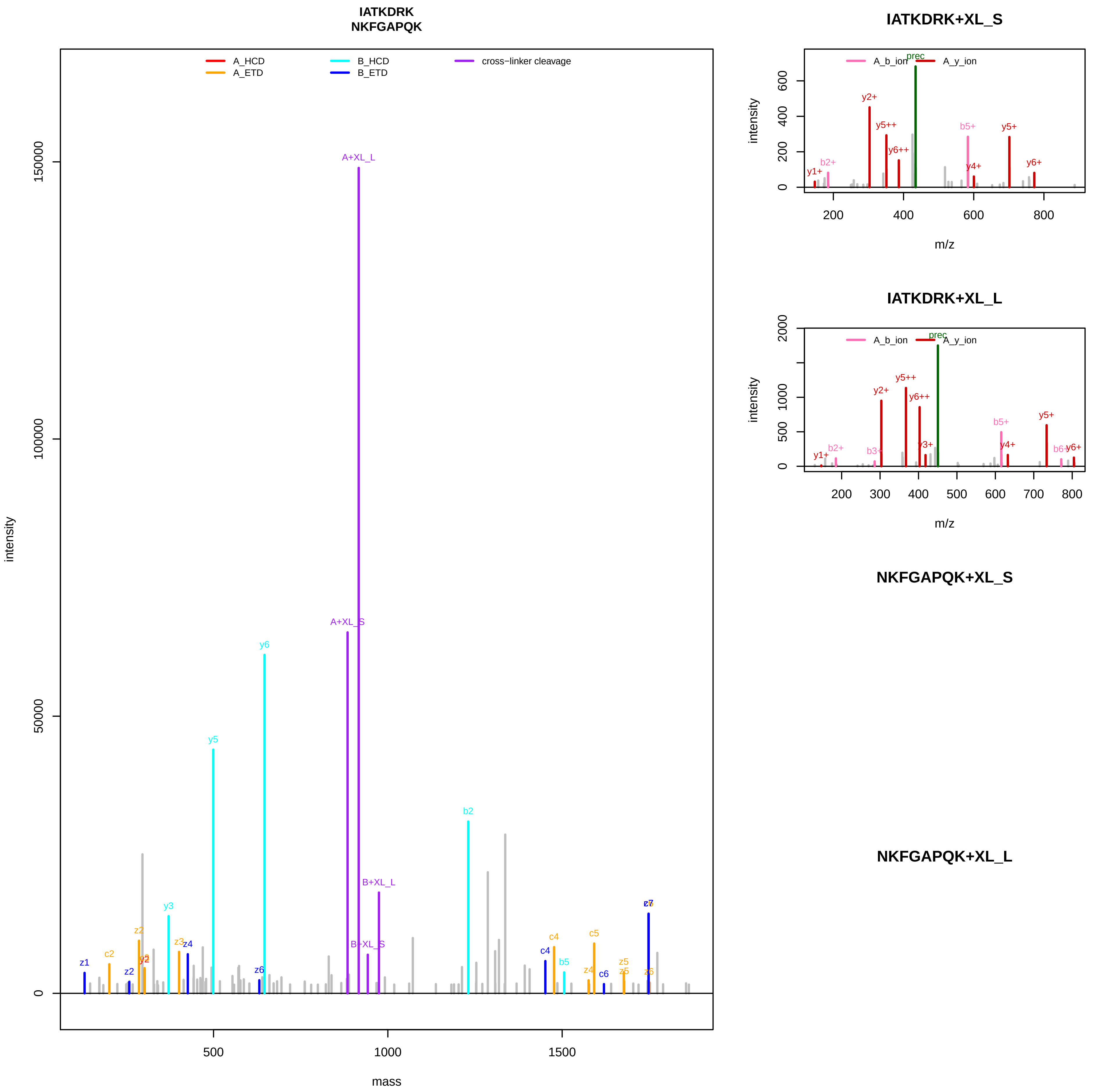
IATKDRK
NKFGAPQK
A_HCD
A_ETD
B_HCD
B_ETD
cross−linker cleavage
intensity
mass
0
50000
100000
150000
500
1000
1500
A+XL_L
A+XL_S
B+XL_L
B+XL_S
y3
y5
y6
b2
b5
z1
c2
z2
z3
z4
z6
c4
c5
c6
z4
z5
z6
z7
IATKDRK+XL_S
A_b_ion
A_y_ion
prec
intensity
m/z
0
200
400
600
200
400
600
800
y1+
b2+
y2+
y5++
y6++
b5+
y4+
y5+
y6+
IATKDRK+XL_L
A_b_ion
A_y_ion
prec
intensity
m/z
0
500
1000
2000
200
300
400
500
600
700
800
y1+
b2+
b3+
y2+
y5++
y6++
y3+
b5+
y4+
y5+
b6+
y6+
NKFGAPQK+XL_S
NKFGAPQK+XL_L
